# Supplementary material for: Recommendations for finite element modelling of nickel-titanium stents—Verification and validation activities
Source: PLoS One. 2023 Aug 9;18(8):e0283492. doi: 10.1371/journal.pone.0283492 (PMC10411813; doi:10.1371/journal.pone.0283492)
Supplement: S1 Appendix — (DOCX) [file pone.0283492.s001.docx]

Appendix and Supporting Information

**Recommendations for finite element modelling of nickel-titanium stents – verification and validation activities**

Martina Bernini ^1,2^, Rudolf Hellmuth ^2^, Craig Dunlop ^2^, William Ronan ^1^, Ted J. Vaughan ^1^

^1^ Biomechanics Research Centre (BioMEC), College of Science and Engineering, University of Galway, Galway (Ireland).

^2^ Vascular Flow Technologies, Dundee (United Kingdom).

# Appendix

**Table A 1**. Literature review of structural analysis of self-expanding nickel-titanium devices for endovascular applications (n/a = not available).

| Reference | Device, FE Model Description and Solver | Input and Output Data | Verification and Validation Activities |
| --- | --- | --- | --- |
| Gökgöl *et al.* (2015) [1] | **Device**: Astron-Pulsar and Astron (Biotronik, Switzerland) for peripheral applications.  **FE Model**: large and complex interactions among components caused discontinuities.  **Solver**: Explicit (Abaqus, Dassault Systèmes Simulia Corp., USA). | **Input Data:**   - ***Geometry***: reconstruction methodology not described. - ***Material***: superelastic behaviour of Nickel-titanium, with parameters provided by the stent manufacturer. - ***Mesh Density***: density n/a; total number of elements of 173,160 and 91,248 for the two devices respectively. - ***Element Type***: linear brick element with reduced integration (C3D8R). - ***Target Time Increment***: 4.0E-06 s.   **Output Data:**   - Radial force curve for mechanical comparison. - Lumen gain and arterial stress after implantation. - Strain distribution in the stent for fatigue analysis. | **Verification**:   - Stent mesh density assessed with a sensitivity analysis that monitored the maximum principal strain until changes less than 2.5% was found. - Target time increment ensured that ratio of kinetic to internal energy was below 5% (quasi-static condition).   **Validation**:   - Artery axial and circumferential behaviour assessed through comparison with published experimental data from uniaxial extension. - Stent mechanical behaviour was compared with radial force data from experiments. |
| Conti *et al.* (2017) [2] | **Device**: PRECISE Stent (Cordis Endovascular, Florida USA) for peripheral applications.  **FE Model**: Nonlinear, large deformation, complex contact (general contact algorithm).  **Solver**: Explicit (Abaqus, Dassault Systèmes Simulia Corp., USA) | **Input Data:**   - ***Geometry***: high resolution micro-CT scan of a real sample. - ***Material*:** superelastic VUMAT (Auricchio & Taylor [3]), with material parameter derived from literature. - ***Mesh Density***: density n/a; total number of element (421,872) and nodes (787,500). - ***Element Formulation***: linear brick element with reduced integration (C3D8R). - ***Target Time Increment***: n/a.   **Output Data:**   - Stent configuration and Von Mises stress distribution for straight and bent leg positions. | **Verification:** n/a.  **Validation:** n/a. |
| Allegretti *et al.* (2018) [4] | **Device**: Two stents featuring a peak-to-valley and a peak-to-peak design for peripheral applications.  **FE Model**: n/a.  **Solver**: ANSYS Mechanical APDL 17.2 (Ansys, Inc.). | **Input Data:**   - ***Geometry***: CAD reconstruction based on measurements from optical analyses. A sub-unit of the stent was modelled (3 and 4 rings for the two devices respectively). - ***Material*:** superelastic material formulation available in the commercial solver; material parameters derived from axial tensile tests on three-wires dog-bone specimens. - ***Mesh Density***: number of elements of 197,000 and 203,000 for the two devices respectively. - ***Element Formulation***: 3D 8-node structural solid element with simplified enhanced formulation. - ***Target Time Increment***: n/a.   **Output Data:**   - Force-displacement curves from axial tensile tests. - Mean and alternating strain in the stent for fatigue life assessment under cyclic loading conditions. | **Verification**:   - Stent mesh density assessed with a sensitivity analysis that monitored the stress and strain at the maximal loaded points of the structures and global reaction forces.   **Validation**:   - Material parameters were extracted from the experimental tensile tests on dog-bone specimen then compared with a FE model of the dog-bone traction. - Stent model validated with a comparison of simulation and experimental results of axial/compression tests on the devices. - Comparison of most critical zone in the stent structure in the simulation and in the experiments under fatigue cycle loading. |
| Lei *et al.* (2019) [5] | **Device**: E-Luminexx ZVL08060 stent (Bard, USA) for peripheral stenting.  **FE Model**: high nonlinearity due to material, geometry and complex displacement-driven contact problem (surface-to-surface contact pair).  **Solver**: Explicit (Abaqus, Dassault Systèmes Simulia Corp., USA). | **Input Data:**   - ***Geometry***: CAD reconstruction based on measurement from high resolution micro-CT scan of a real sample. - ***Material***: superelastic VUMAT (Auricchio & Taylor [3]), with material parameter derived from literature on a different stent design. - ***Mesh Density***: 4 × 4 element density in width and thickness. - ***Element Formulation***: incompatible linear brick elements (C3D8I). - ***Target Time Increment***: n/a.   **Output Data:**   - Contour plot of radial displacement in deployment simulation in the stent and the artery. - Mean and alternating strain in the stent for fatigue analysis under physiological biomechanical conditions. | **Verification**:   - Mesh density sensitivity analysis mentioned but details are not reported. - Selection of appropriate loading rate to ensure the simulation to be quasi-static. Evaluation based on the extraction of fundamental frequencies of stent and artery; addition of damping for energy-absorption.   **Validation**: n/a. |
| Feng *et al.* (2019) [6] | **Device**: Innovative stents designed for iliac vein patented by the authors.  **FE Model**: large-scale deformation, nonlinear problem. The generalized variational principle and the penalty function method were used.  **Solver**: Explicit (Abaqus, Dassault Systèmes Simulia Corp., USA) | **Input Data:**   - ***Geometry***: designed and developed by the authors. - ***Material***: perfectly elastic material fitted to uniaxial tensile test of the Nickel-titanium tube specimen; material parameters not reported. - ***Mesh Density***: n/a. - ***Element Formulation***: n/a. - ***Target Time Increment***: n/a.   **Output Data:**   - Maximum principal strain distribution in stent during radial compression. - Maximum principal strain distribution in stent and Von Mises stress distribution in artery after implantation. - Mean and alternating strain for fatigue analysis. | **Verification**: n/a.  **Validation**: n/a. |
| Shen *et al.* (2020) [7] | **Device**: Prototype with a tapered designed, application n/a (outer proximal diameter of 1.74 mm).  **FE Model**: n/a.  **Solver**: n/a (Abaqus, Dassault Systèmes Simulia Corp., USA). | **Input Data:**   - ***Geometry***: designed by the authors. - ***Material***: superelastic VUMAT (Auricchio & Taylor [3]), with material parameters derived from literature. - ***Mesh Density***: 92,945-105,401 elements. - ***Element Formulation***: quadratic tetrahedral elements (C3D10). - ***Target Time Increment***: n/a.   **Output Data:**   - Von Mises stress distribution in the stent and reaction moment in bending deformation. | **Verification**:   - Mesh density sensitivity analysis mentioned but details are not reported.   **Validation**: n/a. |
| Hung *et al.* (2021) [8] | **Device**: Prototype for thrombectomy with a V-shaped design, closed cells and irregular spikes to engage with the blood clot.  **FE Model**: n/a.  **Solver**: Implicit (Abaqus, Dassault Systèmes Simulia Corp., USA). | **Input Data:**   - ***Geometry*:** designed by the authors. - ***Material***: superelastic UMAT (Auricchio & Taylor [3]), with material parameters derived from literature. - ***Element Formulation*:** not specified for the stent, the blood clot is meshed with incompatible linear brick elements (C3D8I). - ***Target Time Increment***: n/a (implicit was used).   **Output Data:**   - Geometric configuration and plastic strain in the device in the expanded, welded, and crimped configurations. | **Verification:** n/a.  **Validation:**   - *In vitro* experiments addressed, but not compared with the FEA (e.g., *in vitro* test to remove the blood clot, bending tests are performed but not compared with a simulation). |
| Zaccaria *et al.* (2021) [9] | **Device**: ID Branch and ID Cav (ID NEST MEDICAL, Strasbourg, France) for the treatment of May-Thurner syndrome in leg veins.  **FE Model**: Nonlinear model, large deformation, complex interaction (general contact algorithm).  **Solver**: Explicit (Abaqus, Dassault Systèmes Simulia Corp., USA). | **Input Data:**   - ***Geometry***: ID Branch was built using 3D parametric equations, with geometrical parameters evaluated from pictures of the stent in the extended configuration; the ID Cav geometry was built from the drawing for the laser-cut manufacturing process. - ***Material***: material parameters were calibrated based on tensile tests performed on wire specimen provided by the manufacturer. - ***Mesh Density***: 5,548 elements (ID Branch) and 9,243 elements (ID Cav). - ***Element Formulation***: linear beam formulation (B31). - ***Target Time Increment***: n/a.   **Output Data:**   - Force-displacement curve in radial compression test, tensile test and free release test. - Von Mises stresses the assembled devices. - Device configuration, von Mises stress in the device, maximum principal stress and strain fields, and contact pressure in the artery when deployed in a realistic geometry. | **Verification**:   - Mesh refinements (coarse, medium and fine) and element type (linear brick elements with reduced integration C3D8R, beam elements B31) assessed on a single unit (V strut). The comparison took included outputs such as reaction force, Von Mises stress and computational time reported as % error w.r.t. the brick element finer mesh. - Material parameters were calibrated from tensile tests at 25°C and 37°C on wire samples, multi-wire specimens, laser-cut stent struct, compared with FEA on a single cube and the multi-wire specimen. - Friction coefficient effects evaluated on the force-diameter curve. - Butterworth filter was applied to both the experimental and numerical curves to reduce the oscillations caused by the explicit method.   **Validation**:   - Device models and material implementation were validated with radial compression or axial tests by comparing in silico outcomes with experiments (*n* = 3) to ensure the test repeatability). The comparison accounted for force-displacement curve (quantitative) and deformation (qualitatively). Different tests were conceived for the different components (either radial compression or tensile test). The assembled device was tested with axial extraction. - Release and recapture procedure was validated in terms of force-displacement curves from pushing/pulling the hub of the delivery system. - Deployment in a realistic geometry validated in terms device configuration (qualitative). |
| Hejazi *et al.* (2021) [10] | **Device:** Diamond design, Chevron design, Z-design and braided design for the treatment of venous obstruction.  **FE Model**: n/a.  **Solver**: Implicit (Abaqus, Dassault Systèmes Simulia Corp., USA). | **Input Data:**   - ***Geometry***: n/a. - ***Material***: superelastic UMAT based on Auricchio & Taylor [3], Bhattacharya [11], Lagoudas model [12], with material parameter derived from literature. - ***Mesh Density***: elements with characteristic length varying from 0.075 mm to 0.05 mm. - ***Element Formulation***: quadratic brick element with full integration (C3D20). - ***Target Time Increment***: n/a (implicit was used).   **Output Data:**   - Von Mises stress distribution in the device. - Bending force-circumferential displacement curves. | **Verification**:   - Characteristic element length based on a mesh sensitivity analysis (details not reported). - Radial pressure calculated based on an analytical equation was compared with data points corresponding to *in vitro* experiment.   **Validation**: n/a. |
| McKenna *et al.* (2021) [13] | **Device**: Precise Pro (Cordis Endovascular, Johnson & Johnson, USA) self-expanding laser-cut stent with and without polymer covering for biliary or femoropopliteal stenting. Additional stent designs and stiffer polymer covers were considered.  **FE Model**: nonlinear model, large deformation, complex interaction (general contact algorithm), penalty contact method (friction coefficient *µ* = 0.2).  **Solver**: Explicit (Abaqus, Dassault Systèmes Simulia Corp., USA). | **Input Data:**   - ***Geometry***: reconstructed from analysing microscope images with ImageJ image processing software. The repeating unit was drawn, meshed, patterned, and wrapped to create the stent cylindrical geometry. - ***Material***: superelastic VUMAT (Auricchio & Taylor [3]), with material parameter calibrated over radial force and axial compression tests for the stent; elastic plastic constitutive behaviour for the cover (PU-PTFE), whose parameters were derived from uniaxial tests on a strip of the sample. - ***Mesh Density***: 4 × 5 in width and thickness, for a total of approx. 470,000 elements. - ***Element Formulation***: linear brick element with reduced integration (C3D8R) and enhanced hourglass control. - ***Target Time Increment***: n/a.   **Output Data:**   - Force - displacement curve in radial compression and axial compression tests. - Bending configuration (qualitatively). - Max principal stress distribution in the stent and in the polymeric cover. | **Verification:**   - Ratio of kinetic energy to internal energy is kept below 5% after initial contact between the stent and rigid surfaces.   **Validation**:   - Radial compression, axial compression and bending tests, used to calibrate/validate the device and the implementation of Ni-Ti, polymeric cover and their interaction. |
| Luraghi *et al.* (2021) [14] | **Device:** EmboTrap II (CERENOVUS, Galway, Ireland)  for trapping and removing blood clot in case of acute ischaemic stroke in brain arteries.  **FE Model**: self-penalty hard contact.  **Solver**: Explicit (LS-DYNA Release 11.0, ANSYS, Canonsburg, PA, USA). | **Input Data:**   - ***Geometry***: CAD model was analysed to extract centreline of the frame and built the wire model, which was assigned a rectangular cross-section. The cross-section dimensions were measured with a confocal laser scanning microscope. - ***Material***: shape memory material constitutive formulation available in the commercial finite-element solver (Auricchio & Taylor [3]). Ni-Ti material parameters were provided by the manufacturer. - ***Mesh Density:*** characteristic length of 0.2 mm, total amount of elements of 4,353. - ***Element Formulation***: Hughes-Liu beam elements with a rectangular cross section. - ***Target Time Increment***: mass scaling (time step = 5E-07 s) and mass proportional damping factor (10 s¯¹) were used.   **Output Data:**   - Force-displacement curve of axial tensile tests on the device. - Configuration of crimping procedure (qualitative). - Thrombectomy procedure in a U-bend tube, funnel-shaped tube, and patient-specific silicone tube. - Von Mises stress and strain in the blood clot during thrombectomy procedure. | **Verification**:   - Mesh density was assessed with three different grids (average element size of 0.4 mm, 0.2 mm and 0.1 mm). Resultant force and the axial stresses on selected elements in the central part of the device used as monitored variables for the convergence analysis. - Mass scaling and damping were selected such that the ratio of kinetic energy to internal energy is kept below 2%.   The authors published more recently a verification study where mesh density, element formulation and integration were comprehensively addressed [15].  **Validation**:   - Material parameters with axial tensile test on the device level (quantitative). - Thrombectomy procedure in a U-bend tube, funnel-shape tube, patient-specific silicone tube (qualitative). Silicone tube was used for the *in vitro* comparator, in the FE model the vessels were modelled as rigid bodies. |
| He *et al.* (2022) [16] | **Device**: Zilver (Cook, USA), and modified/personalised design.  **FE Model**: contact pair interaction between the stent and the vessel, general hard contact with a friction coefficient *µ* = 0.2.  **Solver**: Explicit (Abaqus, Dassault Systèmes Simulia Corp., USA). | **Input Data:**   - ***Geometry***: not reported for the Zilver, other designs were built by the authors. Plaque model was constructed with from DICOM images of a patient’s femoropopliteal plaque using CT imaging. - ***Material***: superelastic VUMAT (Auricchio & Taylor [3]), with material parameter from literature (different stent design). - ***Mesh Density***: 4 × 4 in width and thickness. - ***Element Formulation***: linear brick elements with reduced integration (C3D8R). - ***Target Time Increment***: 1E-08 s, obtained by selecting a mass scaling factor of 1000, damping coefficient of 1.2 for quadratic bulk viscosity.   **Output Data:**   - Lumen gain and cross-section shape post deployment. - Von Mises stress in the artery (media layer). | **Verification**:   - Mesh density and element type selected according to previous studies of the same authors [17], [18]. Schiavone *et al. (*2016) [17] selected a 4 × 4 mesh density of linear brick elements with full integration and incompatible mode (C3D8I) considering the numerical convergence in terms diameter change, recoiling and stress distribution. Qiu *et al.* (2018) [17], [18] selected a 5 x 6 mesh density of linear brick elements with reduced integration (C3D8R). Noticeably, the mesh density relies on different stent geometries and materials: Xience (Abbott, USA) stent in Co–Cr alloy L605 [17] and Absorb (Abbott, USA) stent in polymeric material (PLLA) [18].   **Validation:** n/a. |

# Supporting Information

## Data Repository

The CAD model of the subunit was uploaded on ZENODO repository (10.5281/zenodo.7622055).

## Comparison Functional Unit and Full Stent

The stent functional unit cell could be employed in some activities to reduce computational costs, provided that quantities of interest (e.g., forces) are properly scaled to be representative of the real device performance. The radial force is obtained from the FEA simulation as the sum of the force recorded at each reference point (RP), as described in **Figure 1**. To make the results consistent with the outcomes obtained from a full device, the radial force predicted by the subunit has to be scaled × 8 (since the unit is repeated 8 times in the circumferential direction), and finally, all the results have to be normalized by the stent axial length (**Figure 1a**) as commonly performed to compare stents of different lengths [19]. The comparison of the FEA among subunit and full stent is shown in **Figure 1b**, where stents have been crimped to a minimum diameter of 2 mm. A good match was found comparing the FEA results, either in terms of global outputs (force, **Figure 1b**) and in terms of local outputs (stress, **Figure 1d**) with minor differences caused by the fact that no boundary conditions were applied to the subunit in the axial (z) direction, while likely in the full model and the real device an additional constrain from the adjacent rings is present. Results overall suggest that upon applying the appropriate scaling factor (to account for the circumferential symmetry) and by normalizing by stent length, the values of force and stresses predicted are within the same range. Finally, from the comparison of the computational time, using the subunit allowed to reduce the computational time of 98% (**Table 1**).

**Table 1**. Comparison of computational time.

| **Computational Time**  [hh:mm:ss] | |
| --- | --- |
| *Subunit* | *Full stent* |
| 00:24:30 | 18:03:58 |


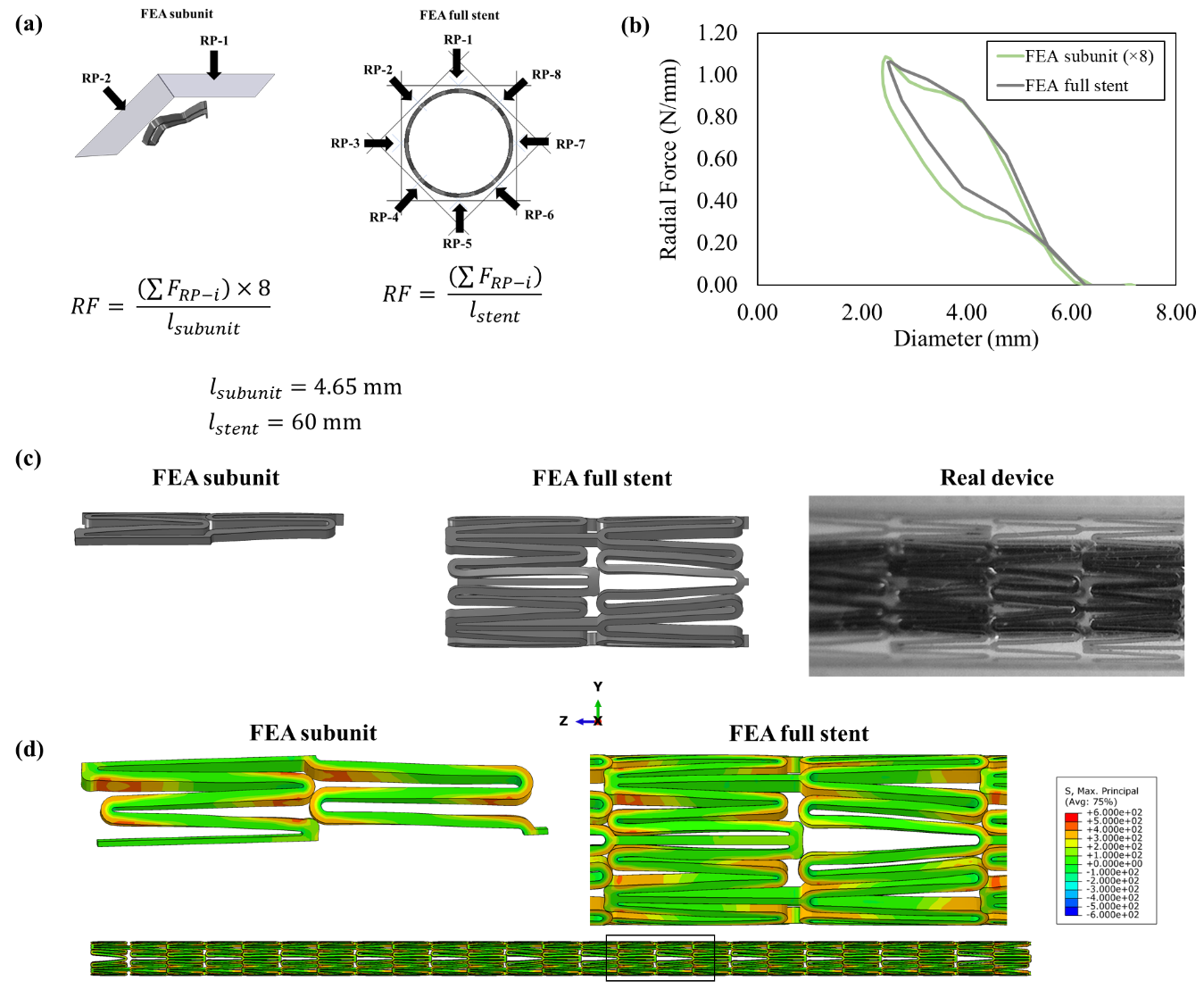


**Figure 1**. Comparison radial force behaviour in the subunit and in the full stent.

# References

[1] C. Gökgöl, N. Diehm, F. R. Nezami, and P. Büchler, “Nitinol Stent Oversizing in Patients Undergoing Popliteal Artery Revascularization: A Finite Element Study,” *Ann. Biomed. Eng.*, vol. 43, no. 12, pp. 2868–2880, 2015.

[2] M. Conti *et al.*, “Patient-specific finite element analysis of popliteal stenting,” *Meccanica*, vol. 52, no. 3, pp. 633–644, 2017.

[3] F. Auricchio, R. L. Taylor, and J. Lubliner, “Shape-memory Alloys: macromodelling and numerical simulations of the superelastic behavior,” *Comput. Methods Appl. Mech. Engrg*, vol. 146, pp. 281–312, 1997.

[4] D. Allegretti, F. Berti, F. Migliavacca, G. Pennati, and L. Petrini, “Fatigue Assessment of Nickel–Titanium Peripheral Stents: Comparison of Multi-Axial Fatigue Models,” *Shape Mem. Superelasticity*, vol. 4, no. 1, pp. 186–196, 2018.

[5] L. Lei *et al.*, “Finite element analysis for fatigue behaviour of a self-expanding Nitinol peripheral stent under physiological biomechanical conditions,” *Comput. Biol. Med.*, vol. 104, no. July 2018, pp. 205–214, 2019.

[6] H. Q. Feng *et al.*, “Finite element analysis and animal test verification of nitinol alloy iliac vein stent performance,” *Proc. Inst. Mech. Eng. Part C J. Mech. Eng. Sci.*, vol. 233, no. 17, pp. 6197–6208, 2019.

[7] X. Shen, J. B. Jiang, H. F. Zhu, Y. Q. Deng, and S. Ji, “Self-Expandable Tapered Stent,” vol. 36, no. 4, pp. 577–584, 2020.

[8] M. Y. Hung, C. K. Yang, J. H. Chen, L. H. Lin, and H. M. Hsiao, “Novel blood clot retriever for ischemic stroke,” *Micromachines*, vol. 12, no. 8, 2021.

[9] A. Zaccaria *et al.*, “Finite Element Simulations of the ID Venous System to Treat Venous Compression Disorders: From Model Validation to Realistic Implant Prediction,” *Ann. Biomed. Eng.*, vol. 49, no. 6, pp. 1493–1506, 2021.

[10] M. Hejazi, F. Sassani, J. Gagnon, Y. Hsiang, and A. S. Phani, “Deformation mechanics of self-expanding venous stents: Modelling and experiments,” *J. Biomech.*, vol. 120, no. 604, pp. 1–30, 2021.

[11] K. Bhattacharya, *Microstructure of Martensite: Why it Forms and how it Gives Rise to the Shape-memory Effect*. Oxford University Press, Oxford.

[12] D. Lagoudas, *Shape Memory Alloys: Modeling and Engineering Applications*. Springer Science and Business Media, 2008.

[13] C. G. McKenna and T. J. Vaughan, “A Computational Framework Examining the Mechanical Behaviour of Bare and Polymer-Covered Self-Expanding Laser-Cut Stents,” *Cardiovasc. Eng. Technol.*, 2021.

[14] G. Luraghi *et al.*, “Applicability assessment of a stent-retriever thrombectomy finite-element model,” *Interface Focus*, vol. 11, no. 1, p. 20190123, 2021.

[15] G. Luraghi, S. Bridio, F. Migliavacca, and F. J. Matas Rodriguez, “Self-expandable stent for thrombus removal modeling : Solid or beam finite elements?,” *Med. Eng. Phys.*, vol. 106, no. January, p. 103836, 2022.

[16] R. He, L. Zhao, V. V. Silberschmidt, J. Feng, and F. Serracino-Inglott, “Personalised nitinol stent for focal plaques: Design and evaluation,” *J. Biomech.*, vol. 130, p. 110873, 2022.

[17] A. Schiavone and L. G. Zhao, “A computational study of stent performance by considering vessel anisotropy and residual stresses,” *Mater. Sci. Eng. C*, vol. 62, pp. 307–316, 2016.

[18] T. Qiu, R. He, C. Abunassar, S. Hossainy, and L. G. Zhao, “Effect of two-year degradation on mechanical interaction between a bioresorbable scaffold and blood vessel,” *J. Mech. Behav. Biomed. Mater.*, vol. 78, no. September 2017, pp. 254–265, 2018.

[19] K. Maleckis *et al.*, “Comparison of femoropopliteal artery stents under axial and radial compression, axial tension, bending, and torsion deformations,” *J. Mech. Behav. Biomed. Mater.*, vol. 75, no. March, pp. 160–168, 2017.
