# Supplementary material for: Recommendations for finite element modelling of nickel-titanium stents—Verification and validation activities
Source: PLoS One. 2023 Aug 9;18(8):e0283492. doi: 10.1371/journal.pone.0283492 (PMC10411813; doi:10.1371/journal.pone.0283492)
Supplement: Attachment — Submitted filename: Response_to_Reviewers.docx [file pone.0283492.s002.docx]

**Response to Reviewers**

The authors thank the reviewers for their valuable comments. We have sought to address the reviewer’s main concerns below that provides additional context to the study. We have considered all issues raised by the reviewers carefully and have outlined, in detail, all changes that we have made to our manuscript in response to their comments.

**Reviewer #1**

**Q1.1)** Explanation to question 3: In order to make all data available I consider that the CAD geometry of the stent should be shared or uploaded in a public repository.

**R1.1)** The authors agree to the request. The CAD model of the subunit was uploaded on ZENODO repository (10.5281/zenodo.7622055) and this information is presented in the ‘Supporting Information’ file.

**Q1.2)** Minor points: There are two references to Figure 5d on lines 287 and 385. However the attatech Figure 5 has only labels a) b) and c).

**R1.2)** The authors corrected the cross references consistently with the figure labelling (Figure5c) at lines 287 and 385.

**Q1.3)** Table 12: Lumen Gain should be defined in the text. In reference 44 the Lumen Gain is defined as a dimensionless quantity.

**R1.3)** The reference 44 defined the lumen gain as the relative increase of lumen area pre- and post- stent deployment. In our studies (section 4.2.1), since we are considering a subunit (with circumferential symmetry) of an idealised circular cross-section, we measured the increase of diameter hereby defined as the difference between the inner diameter (ID) pre- and post- deployment. To avoid misinterpretation, we changed the parameter definition to ‘Diameter Gain’ [mm] and added the equation (DG = IDpost – IDpre) [mm] at line 514. Changes were made across the text for consistency. In our study, we did not normalize the diameter gain by the initial value (IDpre), thus it maintains a unit of [mm]. This choice was taken since the IDpre = 5 mm across all cases. However, we then preferred to compare the DG against the reference value (e.g., the DG obtained with the most refined mesh grid) to provide the relative difference (%).

**Q1.4)** I think that the version code of the Commercial Software Package used for the simulations should be mentioned.

**R1.4)** The version of the software (Abaqus/Explicit v.2017) was added at line 268 in the ‘Methods’ section.

**Q1.5)** Suggestion for COU-1: a verification activity comparing the results of the functional unit (axisymmetric boundary conditions) with the results obtained with the complete geometry of the stent.

**R1.5)** The suggested activity was performed. Previous examples in the literature have taken advantage of stent unit cell to reduce computational costs (1,2), provided that quantities of interest (e.g., forces) are properly scaled to be representative of the real device performance. In our study we compared the FEA prediction of stent behaviour for radial compression (COU-1). The radial force is obtained from the FEA simulation as the sum of the force recorded at each reference point (RP), as described in **Figure 1**. To make the results consistent with the outcomes obtained from a full device, the radial force predicted by the subunit has to be scaled × 8 (since the unit is repeated 8 times in the circumferential direction), and finally, all the results have to be normalized by the stent axial length (**Figure 1a**) as commonly performed to compare stents of different lengths (3). The comparison of the FEA among subunit and full stent is shown in **Figure 1b**, where stents have been crimped to a minimum diameter of 2 mm. A good match was found comparing the FEA results, with minor differences caused by the fact that no boundary conditions were applied to the subunit in the axial (z) direction, while likely in the full model and the real device an additional constrain from the adjacent rings is present. Results overall suggest that upon applying the appropriate scaling factor (to account for the circumferential symmetry) and by normalizing by stent length, the values of force predicted are within the same range.

A statement has been added to the manuscript to indicate that the radial response of the stent sub-unit was quantitatively compared to the full stent to ensure this assumption was valid (see Section 3.3.1, lines 254-255). Also, additional information reported in this answer has been provided in the ‘Supporting Information’ file.

**Figure 1**. Comparison radial force behaviour in the subunit and in the full stent.

**Q1.6)** In Table 7 and Table 8 Chronic Outward Force is tabulated but in Figures 6 and 7 Radial Force along the test is plotted. Is there any way that the values of the Chronic Outward Force appear in the Figures?

**R1.6)** The chronic outward force (COF) represents a specific point extracted on the unloading curve of the radial compression test. It has a clinical significance since it represents the force exerted by the stent onto the vessel, in our study we measured COF at 5mm on the unloading curve, given that the stent is expected to be deployed in a vessel with an inner diameter of 5mm. For clarity, an indication to the COF, Fmax was added in **Figures 6-7-8-9-10** in the manuscript, as follows:

**Figure 2**. On the left, schematic of the parameters evaluates on the radial force curve, Fmax and COF respectively. On the right, the indication added in the results section.

**Reviewer #2**

In the paper, the finite element modeling of Nitinol stents were studied in verification and validation. It's an interesting work and useful for those interted in developing stents. I have some question on the FEM:

**Q2.1)** how to justify one unit cell is good enough to represent the whole stent?

**R2.1)** Similar to our response to Reviewer 1, previous examples in the literature have taken advantage of stent unit cell to reduce computational costs (1,2), provided that quantities measured (e.g., forces) are properly scaled to be representative of the real device performance. In our study we compared the FEA prediction of stent behaviour for radial compression (COU-1). The radial force is obtained from the FEA simulation as the sum of the force recorded at each reference point (RP), as described in **Figure 1**. To make the results consistent with the outcomes obtained from a full device, the radial force predicted by the subunit has to be scaled × 8 (since the unit is repeated 8 times in the circumferential direction), and finally, all the results have to be normalized by the stent axial length (**Figure 1a**) as commonly performed to compare stents of different lengths (3). The comparison of the FEA among subunit and full stent is shown in **Figure 1b**, where stents have been crimped to a minimum diameter of 2 mm. A good match was found comparing the FEA results, either in terms of global outputs (force, **Figure 1b**) and in terms of local outputs (stress, **Figure 1d**) with minor differences caused by the fact that no boundary conditions were applied to the subunit in the axial (z) direction, while likely in the full model and the real device an additional constrain from the adjacent rings is present. Results overall suggest that upon applying the appropriate scaling factor (to account for the circumferential symmetry) and by normalizing by stent length, the values of force and stresses predicted are within the same range. Finally, from the comparison of the computational time, using the subunit allowed to reduce the computational time of 98% (**Table 1**).

Table 1. Comparison of computational time.

| **Computational Time**  [hh:mm:ss] | |
| --- | --- |
| *Subunit* | *Full stent* |
| 00:24:30 | 18:03:58 |

A statement has been added to the manuscript to indicate that the radial response of the stent sub-unit was quantitatively compared to the full stent to ensure this assumption was valid (see Section 3.3.1, lines 254-255). Also, additional information reported in this answer has been provided in the ‘Supporting Information’ file.

**Q2.2)** the stent is not axisymmetric but can be considered as cyclic symmetric (rotational).

**R2.2)** The authors agree with the reviewer. The boundary conditions that were applied in the model are reported below in **Figure 2** and correspond to a **symmetry around the circumferential axis** (a cylindrical coordinate system was used). In detail, on the edges obtained by cut planes in the circumferential direction (θ), symmetrical boundary conditions in (2/θ) have been applied, which comprises:

$$\left\{ \begin{aligned} U_{2}=0 \\ UR_{1}=0 \\ UR_{3}=0 \end{aligned} \right.$$

(1)

Therefore, the description of the boundary conditions has been emended (from *‘axisymmetric’* to *‘symmetric’*) at lines 251-252.

**Figure 2.** Boundary conditions applied in the model.

**Q2.3)** the fig1c is not corresponding to the rectangular region of fig1a.

**R2.3)** For clarity, the subunit in **Fig 1c** was rotated and the axis were updated consistently to the 2D sketch reported in **Fig 1a**. An updated version is shown here in **Figure 3**. The original figure of the manuscript has been updated with the current version of picture (c) here reported.

**Figure 3.** Comparison of the full stent (a) hereby represented with a 2D sketch, and the corresponding subunit selected (c).

**In response to ‘Additional Requirements’ raised by the editorial office**

1) Our file naming meets PLOS ONE’s style requirements.
2) The ethics statement now includes details on informed consent, type of consent (written), reference to the ethics committee.
3) No codes were developed as part of this manuscript.
4) We kindly ask you to amend the financial disclosure in the online submission form as follows:

“*TJV reports financial support was provided by EU Framework Programme for Research and Innovation Marie Sklodowska-Curie Actions. This study is part of the BioImplant ITN project, which has received funding from the European Union’s Horizon 2020 research and innovation programme under grant agreement No 813869 (*[*https://cordis.europa.eu/project/id/813869*](https://cordis.europa.eu/project/id/813869)*). The funders had no role in study design, data collection and analysis, decision to publish, or preparation of the manuscript. This publication reflects only the author’s view, and the REA is not responsible for any use that may be made of the information it contains.* “

5) The updated manuscript now contains the updated acknowledgment section.
6) We have updated the data availability statement in the revised manuscript and made relevant data, as asked by the reviewers, available in a repository. Details of the repository are now available in the Support Information file.
7) A full ethics statement (as per point #2) is now included in the Methods section of the manuscript.
8) Our reference list is complete and correct. One reference (previously (42), Berti et al., at line 228) has been moved to line 265, becoming (48).

**References:**

1.	Berti F, Bridio S, Luraghi G, Pant S, Allegretti D, Pennati G, et al. Reliable Numerical Models of Nickel-Titanium Stents: How to Deduce the Specific Material Properties from Testing Real Devices. Ann Biomed Eng. 2022;

2.	Antonini L, Berti F, Isella B, Hossain D, Mandelli L, Pennati G, et al. From the real device to the digital twin: A coupled experimental-numerical strategy to investigate a novel bioresorbable vascular scaffold. PLoS One. 2021 Jun 1;16(6). Available from: https://pubmed.ncbi.nlm.nih.gov/34086820/

3.	Maleckis K, Deegan P, Poulson W, Sievers C, Desyatova A, MacTaggart J, et al. Comparison of femoropopliteal artery stents under axial and radial compression, axial tension, bending, and torsion deformations. J Mech Behav Biomed Mater. 2017;75(March):160–8. Available from: https://doi.org/10.1016/j.jmbbm.2017.07.017
